# Editorial: Origins and Domestication of the Grape

**DOI:** 10.3389/fpls.2020.01176

**Published:** 2020-07-31

**Authors:** Fabrizio Grassi, Rosa Arroyo-Garcia

**Affiliations:** ^1^ Department of Biology, University of Bari, Bari, Italy; ^2^ CBGP-INIA (Center for Plant Biotechnology and Genomics-National Institute of Agricultural and Food Research and Technology), Madrid, Spain

**Keywords:** domestication, phylogeny, *Vitis*, vitaceae, evolution, wild, hybridization, introgression

Domestication is the most important genetic process of selection driven by humans that transformed wild forms into domesticated crops modifying morphological and genetic traits. The domesticated grapevine (*Vitis vinifera* L.) belongs together with about another 70 species to the *Vitis* genus (Wen et al., 2018) and being one of the oldest and extensively cultivated fruit crops is a fascinating subject for evolutionary studies. Archaeological data suggest that domestication of the grapevine began 6,000–8,000 years ago in the Transcaucasian region, between the Black Sea and Iran, from populations of *V. vinifera* subsp. *sylvestris* (Zohary and Hopf, 1993). Starting from the primary center of domestication, cultivated varieties were spread by humans through Southern Greece to the Mediterranean region. This pattern of domestication has been recently confirmed by molecular data (Myles et al., 2011; De Lorenzis et al., 2019) even if some authors have proposed local and circumscribed secondary domestication events in other areas (Rivera-Nunez and Walker, 1989; Grassi et al., 2003; Arroyo-Garcia et al., 2006; Terral et al., 2010).

During domestication, humans selected for high yield and rapid growth which led to the non-intentional loss of resilience factors. In grapevine, changes in berry size, sugar content and the shift in sexual system from dioecy to self-pollination were essential for domestication. In the light of this, it would be relevant to go back to the wild progenitor, to identify such factors and make them available for resilience breeding. The resilience factors identified can be directly implemented into breeding strategies targeted to prepare European viticulture for the challenges posed by global climate change. Moreover, there are several intriguing unanswered questions regarding where, when, and how domestication took place. Given the difficulty to infer the number of domestication events that could often be conflated with introgression events with local wild populations (Di Vecchi-Staraz et al., 2009; Zecca et al., 2010; Myles et al., 2011; Zhou et al., 2019), adequate genomic studies that involve local cultivar and wild populations are needed to resolve and understand the process of domestication fully.

Nuclear DNA markers have been widely applied to explain the genetic relationships between wild and cultivated grape lineages while the hypervariable regions within plastid intergenic spacers have been used to investigate the maternal lineages involved in the domestication process (Arroyo-García et al., 2002; Grassi et al., 2002; Snoussi et al., 2004; Arroyo-Garcia et al., 2006; De Mattia et al., 2008; Cunha et al., 2009; Zecca et al., 2010; Karataş et al., 2014). Unfortunately, the low number of markers used and the low variability in grapevine plastid genomes greatly limited domestication studies (Zecca et al., 2020). Today, next-generation sequencing technologies are an extraordinary opportunity to access a large amount of genomic information allowing the debate about the origin of the grapevine to be deepened. In this Research Topic, we have collected a series of original papers about the origins of the grape.

Three research articles have evaluated the phylogenetic relationships between wild and domesticated grapevine samples in different areas of the Mediterranean region. Based on extensive taxon sampling, authors have analysed the DNA of wild samples and local varieties distributed in Sicily (De Michele et al.), Tuscany (D’Onofrio) and Portugal (Cunha et al.). The results suggest that, in addition to the primary ancient domestication center additional events of introgression and/or domestication occurred in other centers of diversification. Moreover, D’Onofrio analyzing SSRs and SNPs polymorphisms shows that introgression processes are still ongoing despite the reduction of wild populations. In another paper, Kui et al. have identified some genomic regions under positive selection that may have been artificially selected throughout the grapevine domestication process. The identification of the genomic regions under selection would not only have great significance in the understanding of the evolution of the grapevine but also have a relevant impact, guiding the genetic improvement in breeding programs.

Though the grapevine is recognized as very important economically in the world and widely used to produce wine and table fruit, several wild grapes are documented as a source of resistance or tolerance genes toward both diseases and environmental stresses and as a source of molecules with beneficial proprieties (Töpfer et al., 2011). A hybrid origin has been proposed for several of them (Munson, 1909) and some taxa have been employed to produce rootstocks resistant to pathogens and new cultivars characterized by the good quality of fruit and resistance to biotic and abiotic stress (Tröndle et al., 2010; Zecca et al., 2012; Wen et al., 2018). Even today, new rootstocks characterized by drought and salt tolerances, are being developed. Using phylogenetic and phylogenomic approaches Zecca et al. have explored the level of natural admixture among America grapes identifying hybrid species with their most likely parent species and showing that introgression events in grapes are more frequent than we thought. Moreover, Wen et al. have investigated the origin of the Concord grape, an economically important cultivar in the United States and Canada. The results confirm its hybrid origin, previously proposed by Huber et al. (2016), and permit the identification of *V*. *vinifera* and *V. labrusca* respectively as the maternal and the paternal donors.

On the other hand, rootstocks which escape from cultivation and naturalize in the wild environment can cause severe consequences for the genetic variability and biodiversity of ecosystems (Arrigo and Arnold, 2007). Arnold and Schnitzler presented an ecological and genetic study contributing to further the knowledge about the behavior of American rootstocks (*V. berlandieri, V. riparia, V. rupestris*) outside their native habitats. These exotic taxa show significant potentialities for invasion, mostly *V. riparia* and its hybrids that could be the best candidates for a large-scale invasion of Europe.

We hope that these papers can contribute to the debate about the origins and domestication of grape even though we know that much effort would have to be made mostly regarding the identification of (*i*) genes under positive selection that can be implemented into breeding strategies, (*ii*) the levels and direction of introgression in grapes and in particular in grapevine, and (*iii*) times and locations where the domestication occurred.

## Author Contributions

All authors contributed to the article and approved the submitted version.

## Conflict of Interest

The authors declare that the research was conducted in the absence of any commercial or financial relationships that could be construed as a potential conflict of interest.
